# The contribution of Chinese-educated physicians to health care in the United States

**DOI:** 10.1371/journal.pone.0214378

**Published:** 2019-04-01

**Authors:** Robbert J. Duvivier, John Boulet, Jason Z. Qu

**Affiliations:** 1 Foundation for the Advancement of International Medical Education and Research, Philadelphia, United States of America; 2 Parnassia Psychiatric Institute, The Hague, the Netherlands; 3 Massachusetts General Hospital, Department of Anesthesia, Boston, United States of America; RTI International Metals Inc, UNITED STATES

## Abstract

**Background:**

Migration of physicians has been a cause for global concern. In China, reforms of the higher education and healthcare systems have led to a shortage of postgraduate training positions relative to the number of medical graduates. Medical graduates opt for non-clinical roles or move abroad to pursue further training and practice opportunities. The impact of this physician migration is not known. This study quantifies where Chinese migrant physicians to the U.S. were educated, where they went to practice, and how these trends have changed over time.

**Methods:**

We combined data on physician characteristics from the 2008 and 2017 American Medical Association Physician Masterfiles with demographic information from the Educational Commission for Foreign Medical Graduates. Using a repeated cross-sectional approach, we reviewed the available data, including citizenship at entry to medical school, medical school attended, practice specialty, and practice location.

**Results:**

The number of Chinese-educated physicians (CEPs) to the United States (US) has increased over the past 10 years, from 3,878 in 2008 to 5,355 in 2017 (+38.1%). The majority held Chinese citizenship at entry to medical school (98.4% vs 97.1%) with the remainder being citizens of other East Asian nations. Of the Chinese citizens identified in 2008, 913 (19.3%) attended medical school outside of China; in 2017, 376 (6.7%) attended medical school outside of China, representing a decrease of 58.8%. Overall, in 2017, four Chinese medical schools provided 32.1% of all Chinese-educated physicians in the US. Over 50% of the CEPs were practicing in Internal Medicine, Anatomic/ Clinical Pathology, Anesthesiology, Family Medicine or Neurology. Compared with all IMGs, CEPs are more likely to be Anatomic/ Clinical Pathologists and Anesthesiologists. CEPs were concentrated in several states, including New York, California and Massachusetts. In 2017, a lower proportion of CEPs in the US healthcare workforce were in residency training, compared to 2008 (13.2% vs 22.8%).

**Conclusions:**

Unlike trends from some other South Asian countries, the number of CEPs in the US has increased over the past 10 years. Migration trends may vary depending on citizenship and country of medical school training. The majority of Chinese-educated graduates come to the US from relatively few medical schools. Fewer CEPs currently in residency training might indicate lower success rates in securing GME training in the US.

## Background

China has the world’s largest physician workforce, with 2,508,408 licensed doctors in 2015 [1]. However, with 1.8 physicians per 1,000 population, China has fewer doctors per capita than the Organisation for Economic Co-operation and Development average (3.4 physicians per 1,000 population) [2]. This absolute shortage of physicians is further compounded by maldistribution, characterized by a concentration of human resources in urban areas, with the doctor density in rural areas being half that of the rest of the country [3].

In an attempt to better address the health needs of this populous country, reforms of both the higher education and healthcare systems have taken place over the last two decades [4]. In short, free-standing medical schools were merged into comprehensive universities [5]. The major healthcare overhaul has focused on the expansion of health insurance coverage and standard postgraduate medical education has been implemented [4,6,7].

This, in turn, has created challenges in various parts of the system [8–10]. For example, expansion of medical student enrolments has created downstream bottlenecks with a large pool of graduates compared to existing postgraduate positions [11]. Whilst the average number of graduates per school is 548 [8], these large cohorts have yet to lead to corresponding changes in workforce densities. Indeed, previous research has shown that many graduates do not enter professional practice [3], instead opting for non-clinical roles or moving to careers in a different field.

Some Chinese graduates also chose to leave the country altogether in pursuit of postgraduate training and practice opportunities elsewhere. The impact of the migration of this group is unknown. To address workforce needs, China, like many other countries, cannot afford to lose highly trained practitioners. The first step in assessing the impact of physician emigration from China is to quantify the number of graduates leaving the country and explore any longitudinal trends. The present study provides data on Chinese-educated physicians (CEPs) who have sought educational and practice opportunities in the U.S. These graduates, while supporting the US physician workforce, represent a net loss to the Chinese medical education and healthcare system.

## Methods

We obtained information on all CEPs working in the US from the 2008 and 2017 American Medical Association (AMA) Physician Masterfiles. The AMA Masterfile contains information on physicians practicing in the US, including type of practice and major professional activity. The AMA data also contains information on practice specialty. The physician’s self-designated practice specialty is determined via questionnaire and is available for most physicians. Because many physicians are not certified by one or more of the 24 American Specialty Boards, the self-designated practice specialty, used for our analyses, may not align with Board certification. We used data from physicians who were active, including those who are employed in non-clinical positions such as research, teaching, or administration. More specifically, active included physicians whose major professional activity (MPA) was classified as full-time hospital staff, resident, research, office-based practice, administration, medical teaching, student, other, locum-tenens, and semi-retired. (The category ‘semi-retired’ was not used in 2008). Physicians who could not be classified based on their MPA were not included in the analysis. To be listed in the AMA Masterfile, an International Medical Graduate (IMG) must have started a residency program in the US.

Using a unique identifier, we combined individual AMA records with demographic information available from the ECFMG [12]. ECFMG certification, which includes credential verification and various assessments, is required to be eligible to obtain a residency position in the U.S. At present, these assessments include the United States Medical Licensing Examination (USMLE) Step 1 (Basic Science), Step 2 CK (Clinical Knowledge) and Step 2CS (Clinical Skills). For IMGs, at least 2 years of residency training, and often three, is required to obtain an unrestricted license to practice medicine in any U.S. jurisdiction. As part of the certification process, ECFMG collects demographic information, including citizenship at entry to medical school. Information on medical schools was obtained from the World Directory of Medical Schools [13,14]. For the purpose of this paper, we defined IMGs as individuals who attended medical schools located outside the US or Canada. Physicians who were educated in China, regardless of citizenship at entry to medical school, are referred to as Chinese-educated physicians, or CEPs.

### Anonymity

Part of the process of data merging consisted of using a unique identifier to match individuals across both data sets. The final data set used for analysis was fully anonymized and data cannot be traced back to individual physicians. Inclusion in either data set is done with consent of the individuals involved, in the case of AMA as part of their member application process, and in the case of ECFMG as part of the application for certification. In this application, the candidate must agree to allow their data to be used for research, or their record is not included in any analysis.

### Analysis

Using a cross-sectional approach, we reviewed the available data, including medical school attended. The primary purpose of the investigation was to quantify and characterize the contribution of CEPs to the US physician workforce. A secondary aim was to document, over time (2008 and 2017), how this contribution has changed over the past 10 years. Data analysis was done with SAS v9.4 software.

## Results

### Total number of Chinese-educated Physicians

Between 2008 and 2017, the active physician pool in the U.S. increased from 788,855 to 893,023 (+13.2%). Of all active physicians in the US in 2017, including those in residency programs, administration, and research and teaching positions, 205,773 (23%) are International Medical Graduates (IMGs). IMGs in the U.S. workforce were educated in many countries, including China. Based on the 2008 Masterfile, 3,878 (0.5%) of active physicians had obtained their medical degree in China, of whom 886 (22.8%) were in residency programs. Most (n = 3,851, 99.6%) did not have English as a native language. In 2017, the total number of Chinese-educated physicians increased to 5,355 (+38.1%), representing 0.6% of the active physician workforce. Of these physicians, 705 (13.2%) were in residency programs (a 20.4% relative decrease in total number compared to 2008). Similar to 10 years prior, most (n = 5,312, 99.4%) were non-native English speakers.

The practice type of active physicians in 2008 and 2017 is presented in Table 1. While the number of CEPs in the US physician workforce grew, there were fewer residents in 2017 (n = 730) than in 2008 (n = 892).

**Table 1:** Practice type of active physicians in 2008 and 2017.

	2008 (all)	%	Chinese Educated Physicians	%	2017		Chinese Educated Physicians	%
Practice Type								
Direct Patient Care	639859	81.1	2895	74.7	724970	81.2	4405	82.3
Resident	110052	14.0	892	23.0	126581	14.2	730	13.6
Medical Research	12598	1.6	22	0.6	9522	1.1	27	0.5
Others*	26346	3.3	69	1.7	31949	3.5	193	5.6
Total	788855	100	3878	100	899,023	100	5355	100

*Includes administration, medical teaching, non-patient care, semi-retired and unclassified.

**Fig 1:**
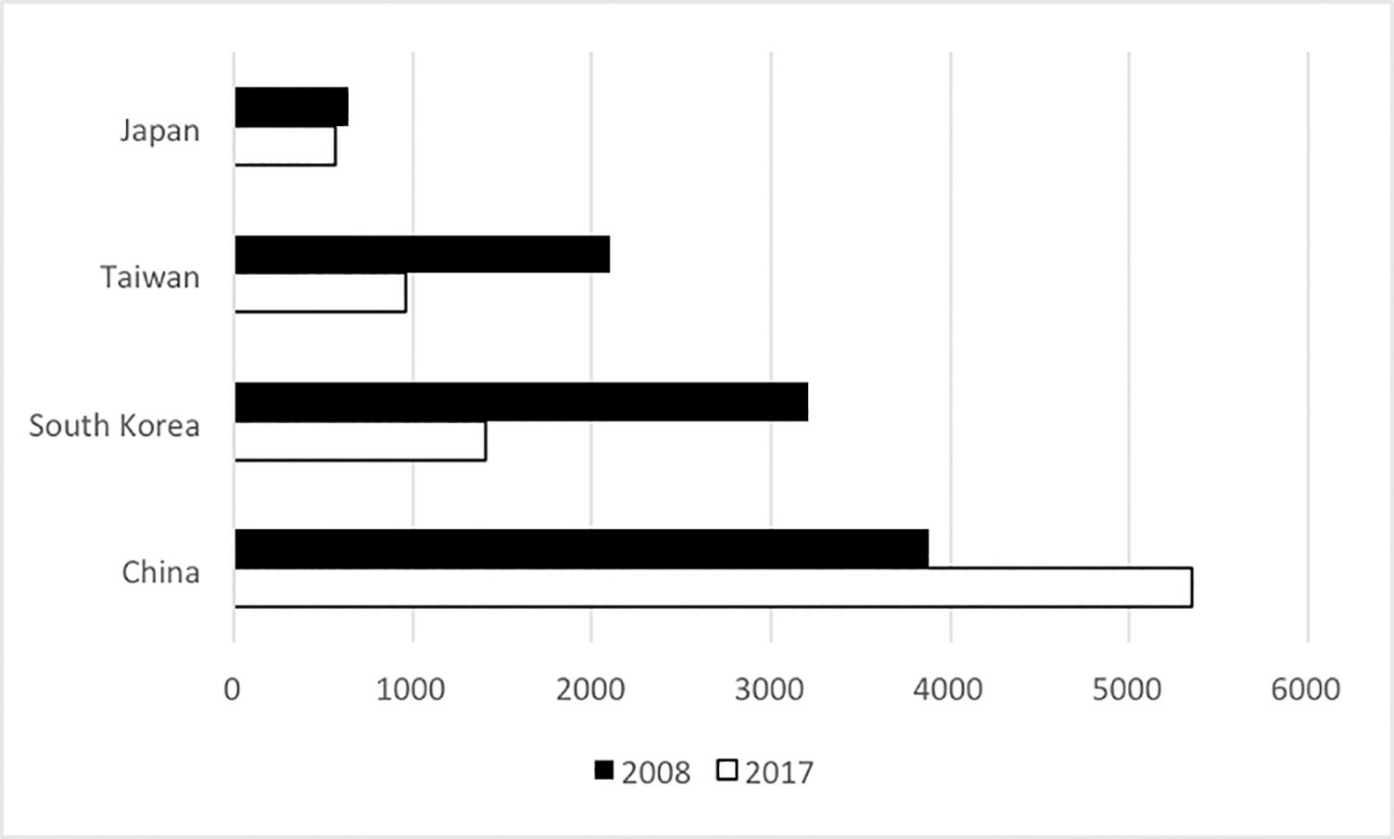
Composition of the Active U.S. workforce from eastern asia (based on country of medical school education).

For comparison, the composition of the active US workforce from Eastern Asia (based on country of medical school training) is presented in Fig 1. There was a 38% increase in the number of Chinese-educated physicians practicing in the U.S. over the 2008–2017 time period. In contrast, there were over 50% decreases in in the numbers of physicians who were educated in South Korea or Taiwan. (Statistics collected by AMA and ECFMG list Taiwan as a separate country. We have followed this convention, which does not imply the expression of any opinion whatsoever concerning the legal status of any country, territory, city or area of its authorities, or concerning the delimitation of its frontiers or boundaries.)

### Citizenship at entry to medical school

From the active physicians who went to medical school in China (n = 3,878), the majority held Chinese citizenship (at entry to medical school). In 2008, this comprised 98.4% of all CEPs, while 0.5% of were listed as Taiwanese, 0.2% from Hong Kong, 0.2% US citizens, and 0.1% from Libya and Nepal each. (Listed are countries with >5 records).

Based on 2017 data, out of 5,355 CEPs 97.1% held Chinese citizenship at entry to medical school, representing an absolute increase of 1,253 individuals or a growth of 32.6%. Other countries of citizenship for CEPs include Nepal (0.7% of all CEPs in 2017), Taiwan (0.4%), India (0.4%), U.S. (0.4%,) Canada (0.4%), Hong Kong (0.1%), Indonesia (0.1%) and Sri Lanka (0.1%).

### Country of medical school

It is also possible for Chinese citizens to obtain the medical degree outside of China. In 2008, 4,727 Chinese citizens (at entry to medical school) were in the U.S. active workforce, representing 2.6% of the IMG workforce. Of these graduates, 3,814 (80.6%) attended medical school in China, and 913 (19.3%) outside of China. Nearly 8% (n = 375) obtained their degree from a medical school located in Taiwan. In 2017, 5,571 Chinese citizens (at entry to medical school) were active as physicians, representing 2.7% of the IMG workforce. Of these graduates, 5,195 (93.3%) attended medical school in China, with 376 (6.7%) outside of China. This represents a decrease of 58.8 compared to 2008. Only 1.5% (n = 81) obtained their medical degree in Taiwan.

### Medical school attended

The top 10 Chinese medical schools for graduates practicing in the US (based on the 2017 AMA Masterfile) are presented in Table 2. In total, 32.1% of all CEPs obtained their degree from one of only four schools and 9 schools have provided nearly half (49.9%) of all CEPs currently practicing in the US. With 187 medical schools in China listed in the World Directory of Medical Schools [13,14], this means 4.8% of schools supplied half of all CEPs and 2.1% supplied one third. Active CEPs graduated from a total of 128 different medical schools in China.

**Table 2:** The top 10 chinese medical schools for graduates practicing in the US (based on the 2008 and 2017 AMA Masterfile).

	2008		2017	
Medical School	N	%	N	%
Peking University Health Science Center	450	11.6	640	12.0
Shanghai Medical University	445	11.5	506	9.5
Zhongshan School of Medicine, Sun Yat-Sen University*			338	6.3
Shanghai Jiao Tong University School of Medicine	197	5.1	233	4.4
Hunan Medical University	171	4.4	218	4.1
Shandong Medical University	141	3.6	189	3.5
China Medical University	102	2.6	188	3.5
Peking Union Medical University	129	3.3	181	3.4
Tongji Medical University	126	3.3	176	3.3
Capital Medical University	122	3.2	149	2.8

*Due to merges and name-changes, earlier graduates were not recorded under this name

### Practice specialties

**Fig 2:**
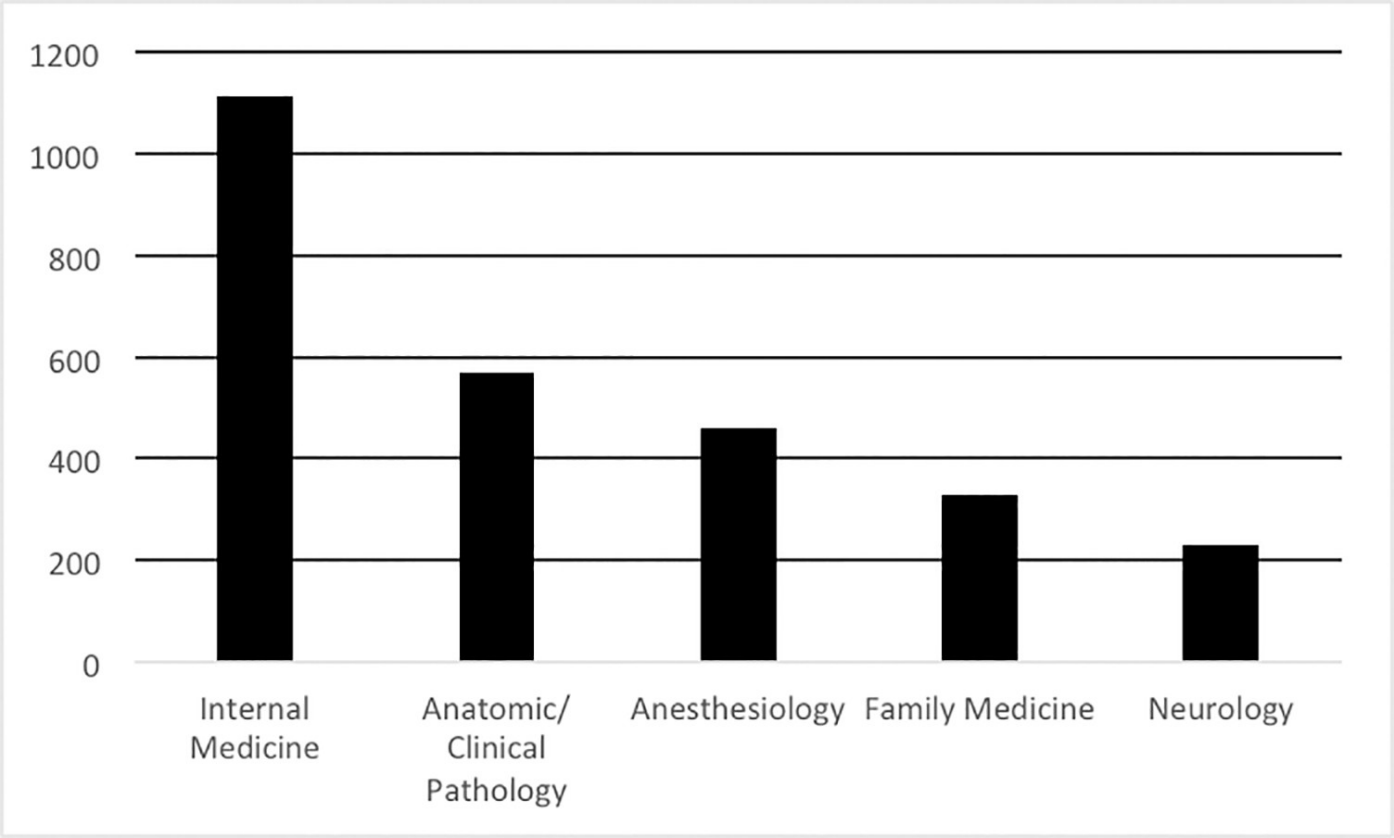
Self-Declared primary practice specialty of CEPs.

The top 5 self-declared primary practice specialties of CEPs in 2017 are shown in Fig 2. Over 50% of the CEPs were practicing in Internal Medicine, Anatomic/ Clinical Pathology, Anesthesiology, Family Medicine or Neurology. For all IMGs, only 3494 (1.7%) indicated that they were Anatomic/ Clinical Pathologists and 8,514 (4.2%) self-declared as Anesthesiologists. For CEPs, 10.8% (n = 571) were Anatomic/ Clinical Pathologists and 8.6% (n = 458) were Anesthesiologists.

### Practice location for CEPs

Chinese-educated physicians practice throughout the US. The location of practice for CEPs (top 10 States), by year (2008, 2017) is presented in Table 3. The summary data shows that CEPs are concentrated in several states, including New York, California and Massachusetts.

**Table 3:** Location of practice for CEPs.

	2008					2017				
	All	IMG	% IMG	China	% China (of IMG)	All	IMG	% IMG	China	% China (of IMG)
California	88518	25569	29.0	573	2.2	100708	21575	21.4	811	3.8
New York	67663	19714	29.1	689	3.4	72193	25215	34.9	850	3.3
Texas	48643	10522	21.6	236	2.2	61232	14564	23.8	351	2.4
Florida	43294	14110	32.6	116	0.8	51994	17797	34.2	194	1.1
Pennsylvania	38776	8497	21.9	212	2.5	41669	9277	22.3	278	3.0
Illinois	34070	10260	30.1	132	1.2	36337	10041	27.6	206	2.1
Ohio	30760	7287	23.7	143	2.0	34677	7689	22.2	205	2.7
Michigan	26797	7523	28.1	123	1.6	29689	8161	27.5	132	1.6
New Jersey	26700	10777	40.4	265	2.5	27411	10499	38.3	300	2.9
Massachusetts	26236	5211	19.9	160	3.1	30015	6220	20.7	240	3.9

## Discussion

To our knowledge, this is the first study to take an in-depth look at Chinese-educated physicians in the US. Before China opened its door to the Western world in the mid 1980s, there were almost no physicians from mainland China entering the US for medical practice. Although based on relatively small baseline numbers, the number of CEPs increased from 2008 to 2017 by 38.1%, far exceeding the percentage growth of all IMGs (13.2%). Our analysis has several implications for both US healthcare and Chinese medical education.

From a US workforce perspective, acceptance into a residency program is the first step in the practice continuum. Our analysis shows an increase in the overall numbers of CEPs in 2017 compared to 2008, which indicates that an increased number of CEPs were successful in obtaining residency positions in the intermediate years, and then stayed in the US. This finding parallels the economic growth of China and the increasing number of students from China studying in the US. The reasons that CEPs leave China are typically classified into push and pull factors [15–17]. Heavy workload, tense patient-physician relations, and pressure to publish research findings may be among the main push factors for Chinese physicians seeking residency training and practice elsewhere [18–21]. Pull factors include better economic conditions and the availability of highly specialized training programs. To better inform workforce policy, the delineation of the specific factors influencing physician emigration from China should be focus of future investigations.

The paradoxical decrease in percentage of all CEPs currently in residency training, compared to 2008 (13.2% vs 22.8%) indicates that fewer Chinese-educated physicians secured GME training in the US. This may be due to a number of factors, including the growth of US medical schools, the requirements for ECFMG certification, and US immigration policy. The pathway to enter US residency training has undergone major changes in the past decades and wider developments, both in undergraduate and graduate medical education, have led to increased competition for spots. These conditions in the US will likely make it more difficult for all IMGs, not just CEPs, to obtain residency positions in the future. Two major developments contribute to this prospect; the establishment of new schools and expansion of class sizes, and no foreseeable increase in GME positions [22,23].

The more competitive nature of US GME, combined with changes in ECFMG certification requirements, could be restricting the pipeline for certain IMG cohorts. The introduction of the ECFMG Clinical Skills Assessment in 1998 [24] and the USMLE Step 2 Clinical Skills component of the licensing exam in 2004 [25], both assessing communication skills, could limit the number of non-native English speakers who are eligible to apply for US residency training. IMG applicants who are successful in securing residency training positions tend to have higher USMLE scores, fewer attempts at ECFMG certification examinations, and speak English as a native language [26]. A key difference between Chinese medical education and major IMG supply countries such as India and Pakistan, is that Mandarin is the only language of instruction in medical schools in China. Classes are given in English only to a minority group of students in few medical schools; notably 6 of the top 10 supplier schools for CEPs have English as their language of instruction. While economic and political factors, including restrictive immigration policies [27,28], may play a role in physician migration, future investigations should look at ECFMG application trends and their relationship to certification examination performance. Since ECFMG certification is required for IMGs to be eligible to enter accredited residency programs in the US, applicant numbers and examination performance can help predict future changes in the composition of the US physician workforce. Since the US is highly reliant on IMGs, these changes could eventually have some negative impact on patient care [29,30].

Another possible reason for the decrease in the number of CEPs in residency in 2017 is the ‘hold’ factor that reflects the unique demographic characteristics in China. Due to socioeconomic and traditional Chinese values and customs, the younger generation is expected to take care of their parents [31]. CEPs who entered the US before 2008 might have siblings in China that their parents can rely on, as opposed to CEPs who were born after 1978 when China instituted the law of ‘one child policy’. Chinese physicians of the ‘single child’ generation might have strong moral reasons to stay in China to take care of their parents [32]. Regardless of the reason for the decrease in Chinese-educated medical school graduates in residency programs, there are likely to be fewer practicing physicians from China in the US workforce, at least in the short-term.

We also found that there are a considerable number of Chinese citizens who obtained their medical degree outside of China. However, based on the more recent data, we found that, proportionately, fewer Chinese citizens obtained their medical degree outside of China. This may be due to a number of factors, including expansion of schools in China over past 10 years and/or an increase in available graduate medical education in China or outside the US. To be included in the AMA Masterfile, an IMG would need to secure a residency position in the US. Chinese-graduates, regardless of where they went to medical school, may be choosing to complete their training in China or elsewhere. Unfortunately, even if an IMG applies for ECFMG certification, and meets the requirements for certification, there is no easy way to track their future movement unless they apply for a US residency position. An in-depth analysis of migration patterns and motivation to study abroad is required to better understand these trends. Although we suspect that this migration for undergraduate medical education is stimulated by a desire to be educated in English, and therefore increase the probability of obtaining GME in the US [33], additional analyses would be required to substantiate this claim. Comparisons between CEPs and Chinese citizens educated outside of China based on ECFMG certification attainment, examination performance, and success in obtaining specialty training would help in understanding the motivations for educational migration. One could also compare USMLE pass rates for graduates of Chinese medical schools and medical schools outside of China that Chinese citizens attend to determine whether there is a quality differential, something that has been observed with graduates from Caribbean medical schools [34,35]. Finally, further exploration of the characteristics of individual CEPs who obtained residency positions in the US can help us better understand career trajectories and predictors for success.

For CEPs specifically, the cost of taking USMLE may also be a deterring factor. Prior to 2008, it was hard for CEPs to take USMLE because there were no practice examination centers in China. Also, the Step 2CS component of USMLE, initiated in 2004, must be taken in the US at one of 6 test centers. Securing the visa and traveling to the US can be both challenging and costly. Nevertheless, over the last decade dedicated private tutoring facilities have sprung up [36]. Whilst the quality of such services has not been studied, and none are endorsed by the sponsors of the USMLE, they indicate a growing interest in pursuing US residency training for Chinese medical graduates.

Chinese-educated physicians represent a fairly small proportion of the active US physician workforce, numbering 5,355 in 2017 (<1.0%). Their numbers have, however, grown by almost 40% since 2008. They represent nearly 4% of all IMGs in California and Massachusetts.

The number of physicians who graduated from Chinese medical schools who entered the US work force is small compared to the combined output of all Chinese medical schools. As a result, the net effect of migration of Chinese-educated physicians on the direct provision of health care in mainland China could be considered to be relatively minor. However, we only quantified migration to the US. Given the globalization of medicine, one might expect that Chinese graduates are also migrating to other countries such as Canada and Australia. Also, it is possible for IMGs to be ECFMG certified and never obtain a residency position in the US. These potential “physician migrants” were not counted, yet may still be a loss in terms of the provision of patient care in China. Finally, one could still argue that having over 450 trained anesthesiologists in the US who graduated from Chinese medical schools represents a fairly significant loss, especially if one considers the number of possible physician-patient interactions this is likely to represent.

Though the loss of medical graduates from China can be construed to be negative, there may also be positive aspects of migration. If physicians return to China following postgraduate education in the US, as has been noted for scientists and entrepreneurs [37,38], the overall effect could be positive. The J1 visa program in the US was specifically developed for this purpose [39]. Unfortunately, while returning CEPs might facilitate uptake of new medical technologies and collaboration between US and Chinese institutions, there are no publicly available data sources to gauge such ‘brain circulation’.

Future research could investigate reverse migration rates, including drivers for such decisions. By studying career trajectories of CEPs we might gain insight in the benefits of pursuing graduate medical education in the US both on individual as well as societal level. Similarly, studies could identify whether returnees continue to practice medicine and explore to what extent their US training has influenced both clinical and research activities.

### Limitations

There are a number of limitations of this investigation. First, we only looked at Chinese-educated physicians at the two specific time points of 2008 and 2017. Second, while the AMA Master file is the best available source for US physician workforce data, it may have its own limitations, including over or under counting physicians in different practice settings [40]. Third, AMA Masterfile does not contain information on the reverse migration of IMGs, physicians who go back to the countries where they attended medicals schools either right after finishing their training or after practicing for some time. Finally, while we documented trends, the cause-effect relationships are not known. For example, we are not able to directly link CEP trends to changes in Chinese medical education standards or the development of residency training programs similar to those in the US [41].
